# Anchored Multiplex PCR Custom Melanoma Next Generation Sequencing Panel for Analysis of Circulating Tumor DNA

**DOI:** 10.3389/fonc.2022.820510

**Published:** 2022-04-12

**Authors:** Russell J. Diefenbach, Jenny H. Lee, Ashleigh Stewart, Alexander M. Menzies, Matteo S. Carlino, Robyn P. M. Saw, Jonathan R. Stretch, Georgina V. Long, Richard A. Scolyer, Helen Rizos

**Affiliations:** ^1^ Macquarie Medical School, Faculty of Medicine, Health and Human Sciences, Macquarie University, Sydney, NSW, Australia; ^2^ Melanoma Institute Australia, The University of Sydney, Sydney, NSW, Australia; ^3^ Department of Medical Oncology, Chris O’Brien Lifehouse, Sydney, NSW, Australia; ^4^ The Faculty of Medicine and Health, The University of Sydney, Sydney, NSW, Australia; ^5^ Department of Medical Oncology, Northern Sydney Cancer Centre, Royal North Shore Hospital, Sydney, NSW, Australia; ^6^ Crown Princess Mary Cancer Centre, Westmead and Blacktown Hospitals, Sydney, NSW, Australia; ^7^ Department of Melanoma and Surgical Oncology, Royal Prince Alfred Hospital, Sydney, NSW, Australia; ^8^ Charles Perkins Centre, The University of Sydney, Sydney, NSW, Australia; ^9^ Department of Tissue Pathology and Diagnostic Oncology, Royal Prince Alfred Hospital and NSW Health Pathology, Sydney, NSW, Australia

**Keywords:** anchored multiplex PCR, melanoma, circulating tumor DNA, targeted sequencing, custom panel, TERT promoter

## Abstract

Detection of melanoma mutations using circulating tumor DNA (ctDNA) is a potential alternative to using genomic DNA from invasive tissue biopsies. To date, mutations in the GC-rich *TERT* promoter region, which is commonly mutated in melanoma, have been technically difficult to detect in ctDNA using next-generation sequencing (NGS) panels. In this study, we developed a custom melanoma NGS panel for detection of ctDNA, which encompasses the top 15 gene mutations in melanoma including the *TERT* promoter. We analyzed 21 stage III and IV melanoma patient samples who were treatment-naïve or on therapy. The overall detection rate of the custom panel, based on *BRAF*/*NRAS*/*TERT* promoter mutations, was 14/21 (67%) patient samples which included a *TERT* C250T mutation in one *BRAF* and *NRAS* mutation negative sample. A *BRAF* or *NRAS* mutation was detected in the ctDNA of 13/21 (62%) patients while *TERT* promoter mutations were detected in 10/21 (48%) patients. Co-occurrence of *TERT* promoter mutations with *BRAF* or *NRAS* mutations was found in 9/10 (90%) patients. The custom ctDNA panel showed a concordance of 16/21 (76%) with tissue based-detection and included 12 *BRAF*/*NRAS* mutation positive and 4 *BRAF/NRAS* mutation negative patients. The ctDNA mutation detection rate for stage IV was 12/16 (75%) and for stage III was 1/5 (20%). Based on *BRAF*, *NRAS* and *TERT* promoter mutations, the custom melanoma panel displayed a limit of detection of ~0.2% mutant allele frequency and showed significant correlation with droplet digital PCR. For one patient, a novel MAP2K1 H119Y mutation was detected in an *NRAS/BRAF/TERT* promoter mutation negative background. To increase the detection rate to >90% for stage IV melanoma patients, we plan to expand our custom panel to 50 genes. This study represents one of the first to successfully detect *TERT* promoter mutations in ctDNA from cutaneous melanoma patients using a targeted NGS panel.

## Introduction

The analysis of circulating tumor DNA (ctDNA) is progressively being integrated into routine clinical care to monitor cancer recurrence, response to therapy, emergence of resistance and to guide therapy (1–3). In melanoma, ctDNA assessment [reviewed in (4, 5)] predicts overall survival of stage IV melanoma patients treated with BRAF and MEK inhibitors or immunotherapy (6–14) and the relapse-free and melanoma-specific survival of patients with high-risk stage III resected melanoma (15–18). ctDNA can also detect the appearance of treatment-resistant melanoma subclones (6), tumor heterogeneity (19), and metabolic tumor burden (20). ctDNA analysis can inform when to cease therapy (21), predicts disease progression after cessation of immunotherapy (22) and differentiates “true progression” from “pseudoprogression” in melanoma patients treated with immunotherapy (23).

To date, a limited number of studies have employed targeted melanoma next generation sequencing (NGS) panels to analyze mutations in ctDNA (24–29). Targeted ctDNA sequencing panels yielded results concordant with other tissue and liquid biopsy approaches (24–29), and detected ctDNA mutations in 52-74% of stage IV melanoma (24, 25, 27) with 0.1-1.0% limit of detection (LOD). A limitation of these ctDNA panels has been the complete inability to detect *TERT* promoter mutations. This is a significant disadvantage because *TERT* promoter mutations are the most frequent recurrent mutations in melanoma, occurring in 34-80% of cutaneous melanomas and are associated with poor survival (30). The most frequent *TERT* mutations C228T (-124 C>T), C250T (-146 C>T) and CC242TT (138/-139CC>TT) (31–42) occur within high GC DNA regions that is difficult to sequence. As a result, most studies continue to rely on droplet digital PCR (ddPCR), which does not have the multiplexing capabilities of targeted NGS panels, to detect *TERT* promoter mutations (24, 25).

In cutaneous melanoma *TERT* promoter mutations commonly co-occur (80-90%) with *NF1*, *BRAF* or *NRAS* mutations, and TERT promoter mutations are also found in 15-60% of *BRAF/NRAS/NF1* WT cutaneous melanoma background (33, 38–40, 43–46). This highlights the need for any melanoma detection assay to include *TERT* promoter mutations in order to maximize detection rates in *BRAF/NRAS* WT patients. The Guardant360 NGS ctDNA assay (Guardant Health, Redwood City, CA, USA) includes *TERT* promoter mutations (47–50), but this pan-cancer panel is not adjustable or specifically tailored for melanoma and requires an allele frequency above 0.25% to detect mutations with 100% sensitivity. We wanted to explore whether anchored multiplex PCR technology (51), which enables the enrichment of target DNA using gene specific primers located at only one end of the DNA, could concurrently detect *TERT* promoter mutations and driver oncogenes in melanoma liquid biopsies. In this proof of principle study we developed a pilot melanoma NGS panel for ctDNA analysis incorporating 15 genes and the *TERT* promoter. The performance of this custom melanoma mutation panel was evaluated in 21 stage III and IV cutaneous melanoma patient blood samples and compared directly to our previous custom melanoma panel which was based on an Ampliseq-HD workflow (25). Our data confirm that anchored multiplex PCR provided a sensitive and specific melanoma liquid biopsy assay that detects common *TERT* promoter mutations. The design of this panel can be expanded and adjusted to incorporate treatment resistance and predictive mutations and is, therefore, particularly valuable in cutaneous melanoma where most patients will ultimately relapse while on treatment with targeted or immune checkpoint therapies.

## Materials and Methods

### Human Melanoma Samples

The fresh-frozen tissue and blood samples from melanoma patients used in the current study were obtained from the Melanoma Institute Australia biospecimen bank with written informed patient consent and institutional review board approval (Sydney Local Health District Human Research Ethics Committee, Protocol No. X15–0454 and HREC/11/RPAH/444). Healthy blood samples were obtained with written informed patient consent and institutional review board approval (Macquarie University Human Research Ethics Committee Protocol No. 52020195621941). The Oncofocus/OncoCarta panels v1.0 (Agena Bioscience, San Diego, CA, USA) or Find IT solid tumor panel (Sonic Genetics, Macquarie Park, NSW, Australia) were used for detection of melanoma-associated *BRAF*, *NRAS*, *KRAS* and *KIT* variants in tissue samples (52, 53). Immunohistochemistry to detect BRAF V600E using VE1 monoclonal antibody (Abcam, Cambridge, UK) was performed as previously described (54).

Blood (10 ml) was either collected in EDTA tubes (Becton Dickinson, Franklin Lakes, NJ, USA) and processed within 4 h from blood draw or Cell-Free DNA collection tubes (Roche, Basel, Switzerland) and processed within 4 days from blood draw. Tubes were spun at 800 g for 15 min at room temperature. Plasma was then removed into new 15 ml tubes without disturbing the buffy coat and respun at 1600 g for 10 min at room temperature to remove cellular debris. Plasma was stored in 1-2 ml aliquots at -80°C.

### Purification of Circulating Free DNA (cfDNA) From Plasma

Plasma cfDNA was purified using the QIAamp circulating nucleic acid kit (Qiagen, Hilden, Germany) according to the manufacturer’s instructions. cfDNA was purified from to 2-5 mL of plasma. cfDNA was subsequently quantified using a Qubit dsDNA high sensitivity assay kit and a Qubit fluorometer 3 (Life Technologies, Carlsbad, CA, USA), according to the manufacturer’s instructions.

### Purification of DNA From Melanoma Cell Lines

Short term cultures (1-2 weeks) of melanoma cell lines were maintained in Dulbecco’s Modified Eagle media supplemented with 10% heat inactivated fetal bovine serum (Sigma-Aldrich, St Louis, MO, USA), 4 mM glutamine (Sigma-Aldrich, St Louis, MO, USA), and 20 mM HEPES (Sigma-Aldrich, St Louis, MO, USA), at 37°C in 5% CO_2_. Spent medium (4 ml) was harvested 48-72 h after splitting of cells. Medium was centrifuged at 800 g for 15 min and supernatant transferred to a new tube and spun at 1600 g for 10 min. Double spun supernatant was then aliquoted into cryovials and stored at -80°C. DNA was subsequently extracted from harvested medium supernatant as described for plasma cfDNA.

### Custom Melanoma Gene Panel for Targeted NGS of cfDNA

A made-to-order melanoma gene panel consisting of individual forward and reverse primers was obtained from ArcherDX a subsidiary of Invitae (San Francisco, CA, USA). The panel covers nucleotide variants which give rise to melanoma-associated amino acid changes across 15 gene targets (42, 55–58), as well as melanoma-associated nucleotide variants in the promoter region of the *TERT* gene (36, 59) (**Table S1**).

The targeted NGS workflow was based on anchored multiplex PCR (AMP) (51). This consists of the use of anchored nested gene specific primers coupled with universal primers in two rounds of PCR amplification. NGS library preparation and sequencing workflows were according to the manufacturer’s protocols (ArcherDX Liquidplex protocol for Illumina version LA090.2) with panel-specific volumes and cycling conditions according to the manufacturer’s product insert (ArcherDX Liquidplex Macquarie Melanoma version LA771.1). For the second PCR reaction the number of cycles was reduced from 20 to 19.

Library QC and sequencing was performed by the Australian Genome Research Facility (AGRF) Sydney node (Westmead, NSW, Australia). Library quality was assessed using a high sensitivity D1000 screen tape on an Agilent Tapestation 2200 (Agilent, Santa Clara, CA, USA). Individual libraries were quantified using an NEBNext Library Quant Kit for Illumina (NEB, Ipswich, MA, USA) using a CFX384 Real-Time System (Bio-Rad, Hercules, CA, USA). Library concentrations were calculated using a size of 150 bp and subsequently pooled to 4 nM. The final library concentration used for sequencing was 18 pM and included 10% PhiX. Sequencing was performed using a MiSeq reagent kit V3 (600 cycle) (Illumina, San Diego California) run as 300 cycle (150 bp PE) on a MiSeq instrument (Illumina, San Diego California).

Analysis of fastq sequencing files was performed using Archer suite analysis version 6.2.7 at https://analysis.archerdx.com according to the manufacturer’s protocol (Archer Analysis 6.0 User Manual). The detection of background sequencing noise was performed using a normal data set consisting of cfDNA from three healthy controls. For SNP/indel detection the thresholds included: AO ≥ 5; UAO ≥ 3; gnomAD AF ≤ 5%; AF ≥ 95MDAF. Rather than setting an average static background (cutoff) noise profile across a panel Archer analysis establishes a position-specific background noise profile for a panel, based on a normal data set, and this information is used to determine the 95MDAF for every potential variant covered by the panel. Thus, 95MDAF is a function of the sequencing coverage of the variant position and the likelihood of that variant appearing as a result of noise. This position-specific value will therefore differ between variants within the same sample and for the same variant across different samples depending on the sequencing coverage (Invitae 95MDAF technical note APM059.A).

- Alternate observation (AO)=Total number of reads that support the alternate allele.
- Unique alternate observation (UAO)=Total number of unique start sites represented by all the alternate reads that intersect the variant.
- Allele fraction (AF)= Reads that support the alternative allele (AO/DP).
- Depth (DP)= The total high quality unique molecule depth covering the variant.
- gnomAD AF =The frequency of the allele called at this locus, from gnomAD global population (60) (https://gnomad.broadinstitute.org).
- 50 Minimal detectable allele fraction (50MDAF)= The AF at which we would consider a variant significant (i.e., above the background noise) given the provided Normal Data Set and taking all consensus reads into account. If the true AF in the sample is at least this, and this identical experiment were run multiple times, 50% of the time there would be sufficient signal to capture this variant.
- 95 Minimal detectable allele fraction (95MDAF) = The AF at which we would consider a variant significant (i.e., above the background noise) given the provided Normal Data Set and taking all consensus reads into account. If the true AF in the sample is at least this, and this identical experiment were run multiple times, 95% of the time there would be sufficient signal to capture this variant.
- Read or unique fragment = Deduplicated consensus read (i.e. molecular bin) having the same unique molecular barcode.

### ddPCR Analysis

The copy number of ctDNA per μl and MAF was determined using the QX200 ddPCR (Bio-Rad, Hercules, CA, USA) as previously described (7). The amount of input DNA template varied for plasma cfDNA while for melanoma cell lines 5 ng of DNA was used for ddPCR. Cancer-associated BRAF V600E and NRAS Q61K/L/R mutations were detected using ddPCR mutation detection assays (Bio-Rad, Hercules, CA, USA). *TERT* promoter mutations -124 C>T and -146 C>T were identified using ddPCR expert design assays dHsaEXD20945488 (*TERT* C228T_88) and dHsaEXD85215261 (*TERT* C250T_88) (61) (Bio-Rad, Hercules, CA, USA), according to the manufacturer’s instructions. The *TERT* promoter assays were optimized by inclusion of 200 μM 7-deaza-dGTP (New England Biolabs, Ipswich, MA, USA), as previously described (25, 62). The DNA copy number/μl for mutant and wild-type circulating DNA species was determined with QuantaSoft software version 1.7.4 (Bio-Rad, Hercules, CA, USA) using a manual threshold setting. The minimum number of positive droplets for calling a mutation was set at two.

### Statistical Analysis

Pearson correlation coefficient analysis and generation of violin plots was performed using Graphpad Prism version 9.1.2.

## Results

### Cohort and Sample Characteristics

A total of 19 cutaneous melanoma patients and three healthy controls were recruited between August 2015 and May 2021. Of the 19 melanoma patients, 2 had blood samples collected at 2 timepoints making a total of 21 melanoma samples for NGS analysis. Of the melanoma patients, 4/19 (21%) had stage III melanoma with a median age of 73 years (63–83) and 15/19 (79%) had stage IV melanoma with a median age of 65 years (30–88) (**Table 1**). The median age of the healthy control cohort was 39 years (range 29-54) and consisted of 2 females and one male.

**Table 1:** Patient characteristics.

Clinical Characteristics	Stage III patients (n = 4)	Stage IV patients (n = 15)
Age – median (range)	73 (64–84)	65 (30-88)
Sex – no. (%)		
Male	2 (50)	13 (87)
Female	2 (50)	2 (13)
AJCC tumor stage (89) – no. (%)		
M1a or M1b	NA	5 (33)
M1c	NA	5 (33)
M1d	NA	5 (33)
Mutation – no. (%)		
BRAF V600	0 (0)	8 (53)
BRAF non-V600	0 (0)	1 (7)
NRAS	2 (50)	4 (27)
BRAF/NRAS WT	2 (50)	2 (13)
Timing of blood draw*	(n = 5)	(n = 16)
Pre (treatment naïve) or at time of treatment progression	4 (80)	13 (81)
EDT (within 3 weeks)	1 (20)	3 (19)

*One patient from stage III cohort and 1 patient from stage IV cohort had 2 samples. EDT patients did not respond (no complete or partial responders) to treatment.

AJCC, American Joint Committee on Cancer; EDT, early during therapy; NA, not applicable.

Tissue mutation analysis was available for all patients: 8/19 (42%) had a BRAF V600 mutation, 1/19 (5%) had a BRAF non-V600 mutation, 6/19 (32%) had an NRAS Q61 mutation and 4/19 (21%) patients were BRAF/NRAS wild type (**Table 1**).

### Evaluation of the Performance of the Custom Melanoma ctDNA Panel

Based on the 15 gene targets (**Table S1**) our custom melanoma ctDNA panel was predicted to cover 66% of cutaneous melanoma patients’ mutations [skin cutaneous melanoma TCGA dataset (45, 46)], 88% of uveal melanoma patients’ mutations [uveal melanoma TCGA dataset (45, 46)] and 24% of acral melanoma patients’ mutations [acral melanoma TCGA dataset (45, 46)]. For cutaneous melanoma the combination of BRAF V600 and NRAS Q61 mutation targets in the custom panel was predicted to cover 60% of patients [skin cutaneous melanoma TCGA dataset (45, 46)]. Addition of *TERT* promoter mutation targets in the custom panel was predicted to further increase coverage by ~15% in those cutaneous melanoma patients that are NRAS or BRAF WT (33, 38). In contrast for uveal melanoma *TERT* promoter mutations are extremely rare (63, 84). For this reason, we focused only on cutaneous melanoma when testing the panel. The custom panel design included several gene targets that were not included in our previous Thermofisher custom panel design (25) and several gene targets not included in the commercial Guardant360 pan-cancer panel (**Table S1**).

**Figure 1:**
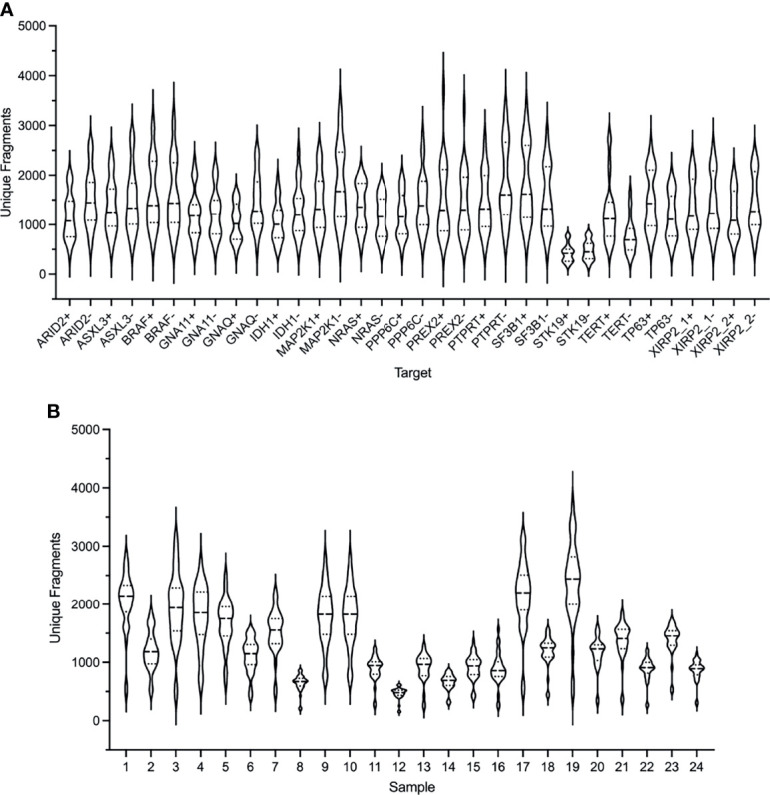
Performance of the ArcherDX custom melanoma ctDNA panel. **(A)** Distribution of unique fragments for each gene specific target in the custom melanoma panel based on sequencing of 24 samples. For each gene target (see **Table S1** for targets) both + and – DNA strand generated fragments are shown. **(B)** Distribution of unique fragments obtained for each sample based on the 34 targets shown in **(A)**. Samples 1-21 are melanoma patients and 22-24 are healthy controls. Next generation sequencing (NGS) libraries were generated using an ArcherDX Liquidplex NGS workflow followed by Illumina MiSeq sequencing. Unique fragments were defined as deduplicated consensus reads having the same unique molecular barcode. Violin plots show median and SD.

The performance of the custom melanoma ctDNA panel was initially evaluated based on unique amplification fragments generated from each forward (+) and reverse (-) gene specific primer (with the universal primer) for each of the 34 target regions (**Figure 1A**). Overall, for the 24 samples analyzed, made up of 21 melanoma patient samples and 3 healthy controls, the mean coverage (based on unique fragments) was similar for the majority of fragments (range 423-1831 unique fragments across 34 target regions). The lowest mean unique fragment count was observed for both fragments covering the *STK19* gene target (STK19- and STK19+ 423 and 478 fragments, respectively), and the *TERT* promoter primers produced 1250 and 768 unique fragments for the TERT+ and TERT- primers, respectively (**Figure 1A**).

Although each sample NGS library passed QC (as described in material and methods) and was sequenced at the same final concentration, there was variation in the median unique fragment count across the 34 amplified target regions for each sample (**Figure 1B**). This did not predict the ability to detect ctDNA mutations as the 7 mutation negative samples (7, 8, 12, 15, 17, 18, 20 and 21) had a median unique fragment count ranging from low to high (**Figure 1A**: median unique fragment count values of 479-2194). Further, this variation in the median unique fragment count was not reflected in the raw paired end reads obtained for each sample which were generally similar (median 654,127 reads) with the exception of sample 7 which had 1,0297,547 raw paired end reads (**Figure S1**). Although sample 7 had the highest paired end reads (**Figure S1**), it did not have the highest mean unique fragment count (**Figure 1B**). Variation in sample performance was not due to the quantity of cfDNA template. For 20 of 24 samples the input was 20 ng while for the remaining samples 10, 20, 22 and 24 the input range was 11.9-15.9 ng. Those samples with the lowest cfDNA input did not yield consistently low median unique fragment counts (**Figure 1B**). Variations in the size distribution of ctDNA may account for variation in sequencing performance. It has been shown that enrichment of ctDNA in the size range 90-150 bp from patients with melanoma improves ctDNA detection by sequencing (64).

### Identification of Melanoma BRAF and NRAS Mutations Using the Custom Melanoma ctDNA Panel

**Figure 2:**
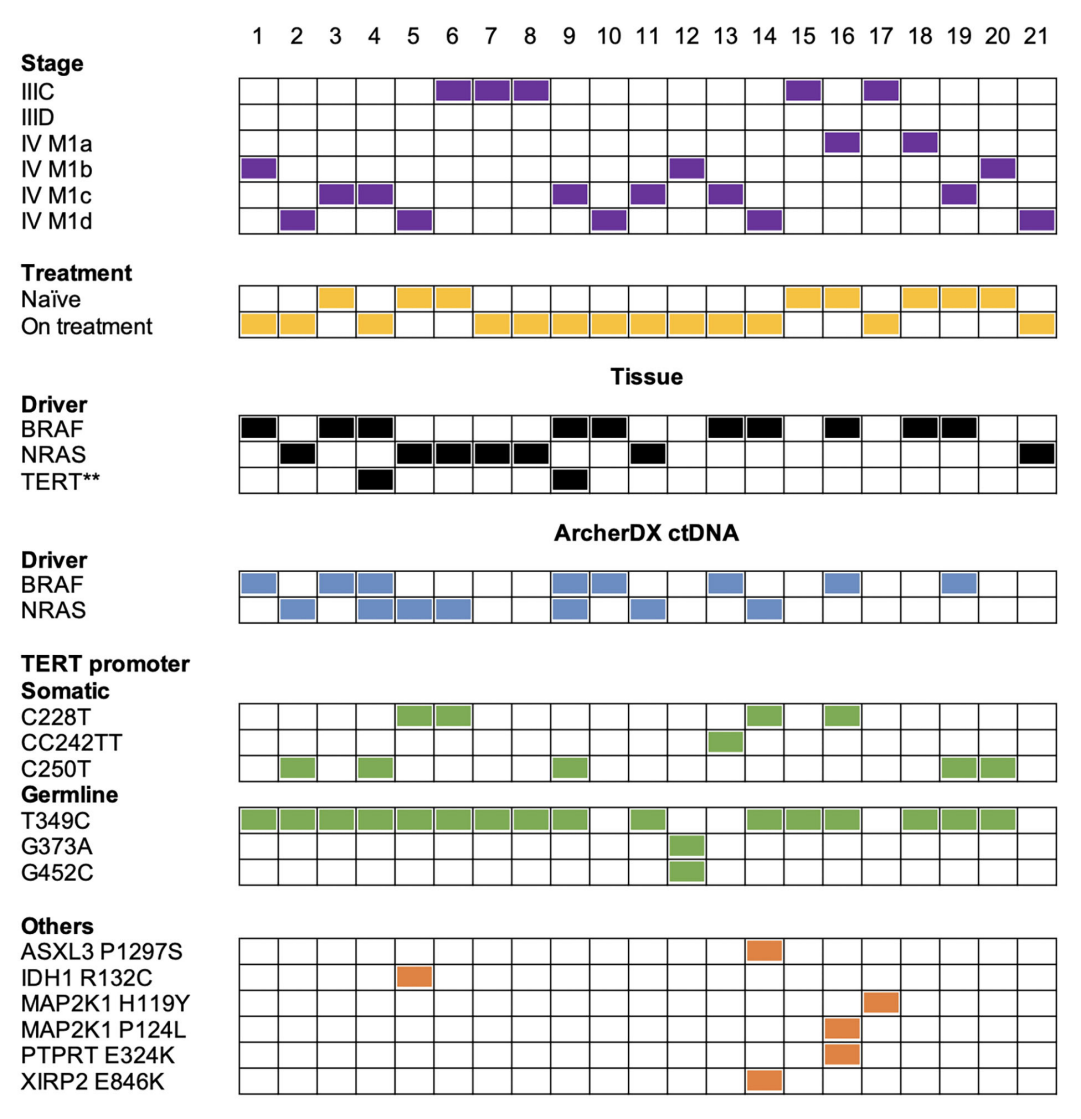
Summary of the melanoma mutation profile identified across the 21 melanoma patient samples from a cohort of 19 melanoma patients. Comparison of melanoma stage (purple boxes), treatment (yellow boxes), tissue driver mutations (black boxes) versus mutations detected with the ArcherDX custom melanoma ctDNA panel including driver mutations (blue boxes), *TERT* promoter somatic mutations or germline variations (green boxes) and other cancer-associated mutations (orange boxes). Numbers represent sample number. For further details on specific gene variations identified and patient details refer to **Table S2**. Samples 4 and 9 are derived from a single patient at two time points. Samples 7 and 8 are derived from a single patient at two time points. **Mutation data from a cell line derived from patient tissue collected 12 months after liquid biopsy samples.

The ArcherDX custom melanoma ctDNA panel detected 13/17 (76%) patients with *BRAF-* or *NRAS-*mutant melanoma. This included 12/14 (86%) stage IV and 1/3 (33%) stage III melanoma patients (**Figure 2**). Liquid biopsies revealed an additional NRAS Q61R-driver mutation in the BRAF V600-mutant samples 4 and 59 from the same patient, and both NRAS mutations were at less than 1% allele frequency compared to the ~30% BRAF mutation frequency (**Tables S2**, **S3**). Initially, only tissue immunohistochemistry was used to detect BRAF V600E (65) in these samples. Subsequently, ddPCR analysis of a patient derived melanoma cell line (corresponding to a timepoint 12 months after liquid biopsy samples 4 and 9) confirmed the BRAF V600E mutation but not the NRAS Q61R mutation (ddPCR data not shown). The negative NRAS Q61R signal is likely due to the time point difference from liquid biopsy samples and the fact this was a selected cell population highlighting the subclonal nature of the NRAS mutation. Although, oncogenic *BRAF* and *NRAS* mutations are usually mutually exclusive (66), they co-occur in approximately 7% of untreated melanoma and *NRAS* mutations confer resistance in 27% of BRAF^V600^-mutant melanoma patients who progress on combination BRAF and MEK inhibitor therapy (67). It is worth noting that this patient (corresponding to samples 4 and 9) had prior combination dabrafenib and trametinib (combi-DT) treatment. The fact that two liquid biopsy samples collected from the same patient at different times displayed identical BRAF/NRAS mutation profiles provides confidence that these mutation data are accurate.

Mutations detected in plasma and tissue were concordant in 16/21 patients (76%) and included 12 *BRAF*/*NRAS* mutation positive and 4 *BRAF/NRAS* mutation negative patients (**Figure 2**). Five samples (samples 7, 8, 14, 18, 21) had a detectable driver mutation in the tissue that was not identified in the liquid biopsy (**Table S2**). We confirmed in samples 7, 8 and 18 that the driver mutation could not be detected in ctDNA using single molecule ddPCR (**Table S3**). Analysis of an NRAS Q61R-mutant NM47 melanoma cell line (68) confirmed that the Q61R ddPCR signal for patient-matched stage III samples 7 and 8 consists of low droplet counts with low intensity (**Figure S3**). Thus, we cannot confidently detect the NRAS Q61R mutation in these two samples by ddPCR or sequencing. ddPCR was unable to detect a BRAF V600E mutation in sample 18 (**Table S3**). Sample 14 had a BRAF G469E mutation which was not covered by our custom ArcherDX panel (**Table S1**). BRAF G469E is a deactivating BRAF mutation that promotes melanomagenesis through oncogenic NRAS (45, 46, 69). This patient has the less common NRAS G60V detected in the liquid biopsy (**Table S2**). This NRAS mutation [TCGA dataset (45, 46)] is predicted to be a driver mutation (COSM4606360). For sample 21 no remaining ctDNA or plasma was available for ddPCR.

### Identification of Melanoma TERT Promoter Mutations Using the Custom Melanoma ctDNA Panel

In contrast to our previous NGS study using a Thermofisher custom melanoma ctDNA panel (25), the ArcherDX custom melanoma ctDNA panel detected *TERT* promoter mutations in ctDNA. Cancer-associated *TERT* promoter mutations (C228T, CC242TT and C250T) (36, 59) were identified in 10/21 (48%) of melanoma samples (**Figure 2**). Of these 5/10 (50%) were C250T, 4/10 (40%) were C228T and 1/10 (10%) was CC242TT. For patient ID 4, corresponding to samples 4 and 9, we did confirm *TERT* promoter mutation C250T in a cell line derived from tissue of the same patient collected 12 months after liquid biopsy using ddPCR (**Figure 2**; ddPCR data not shown). This distribution of *TERT* promoter mutations aligns with previous studies on melanoma (33, 38). Co-occurrence of *TERT* promoter mutations with either BRAF or NRAS mutations was found in 9/10 (90%) of the samples. The detection of a *TERT* C250T mutation in sample 20 (**Figure 2**), which was BRAF and NRAS mutation negative based on both the tissue panel and custom panel (**Table S2**), increased the overall detection rate for the custom panel from 13/21 (62%) to 14/21 (67%) (based on *BRAF/NRAS*/*TERT* promoter).

Although unique fragment coverage for *TERT* promoter centered around cancer-associated *TERT* promoter mutations, the nature of anchored multiplex PCR did result in coverage beyond the target region albeit with lower read depth (**Figure S2**). Therefore we were able to detect a number of previously described germline *TERT* promoter variations (70) including T349C (rs2853669), G373A (rs35226131) and G452C (rs35161420) at MAFs ranging from 38-99% (**Figure 2** and **Table S2**). None of these variations were present in the 3 healthy controls (data not shown). Of these previously described germline variations, T349C was found in 14/21 (67%) patients while G373A and G452C co-occurred in 1/21 (5%) patients (**Figure 2**). A previous study found *TERT* T349C to be present in 52% of melanoma cell lines (43). The high frequency of T349C also aligns with previous studies on other cancers (70–72). All the *TERT* promoter mutations C228T and C250T and 14/17 (82%) *BRAF/NRAS* mutations co-occurred with the germline *TERT* promoter variant T349C (**Figure 2**). There was no clear relationship between either melanoma stage or *BRAF/NRAS* mutation status and the presence of *TERT* promoter variant T349C (**Figure 2**).

### Identification of Other Melanoma Cancer-Associated Mutations Using the Custom Melanoma ctDNA Panel

Several other cancer-associated mutations were identified in 4/21 (19%) patients using the ArcherDX custom melanoma ctDNA panel (**Figure 2**). Of the BRAF and NRAS mutation negative samples, the custom panel was able to detect a MAP2K1 H119Y mutation in sample 17 (**Figure 2**). The patient, who was BRAF V600 WT, had been treated for 1 week with pembrolizumab and subsequently maintained persistently detectable disease. This mutation has been recently proposed to be a non-hotspot *MAP2K1* mutation which activates the ERK pathway (73) and therefore could be targeted with an ERK inhibitor (74). The remaining mutations did not increase detection coverage of the custom panel as they all co-occurred with either BRAF or NRAS driver mutations (**Figure 2**). Of note, PTPRT E324K, along with MAP2K1 P124L, was detected in treatment naïve sample 16 which also harbored a BRAF V600K mutation (**Figure 2**). In melanoma, mutations in PTPRT, such as E324K, which create neoepitopes, may be associated with better outcomes for patients on immunotherapy (75). This may have been an option for this patient who subsequently had a partial response to combination BRAF/MEK inhibition with the presence of MAP2K1 P124L presumably contributing to resistance (76, 77). For sample 14, ASXL3 P1297S and XIRP2 E846K were detected in an NRAS G60V background which was also BRAF G469E (based on tissue biopsy) (**Figure 2** and **Table S2**). Given the MAF of ASXL3 P1297S was 55.88% and this mutation is not a previously identified melanoma-associated mutation (45, 46), suggests that it may be a germline polymorphism or a result of clonal hematopoiesis, which has been noted for mutations in the related protein ASXL1 (78). Sample 5 had an NRAS Q61 and IDH1 R132 mutation in the liquid biopsy and the co-occurrence of these mutations (**Figure 2**) is significant (p<0.001) based on the skin cutaneous melanoma TCGA database analysis of mutual exclusivity (45, 46).

### Sensitivity of the Custom Melanoma ctDNA Panel

**Figure 3:**
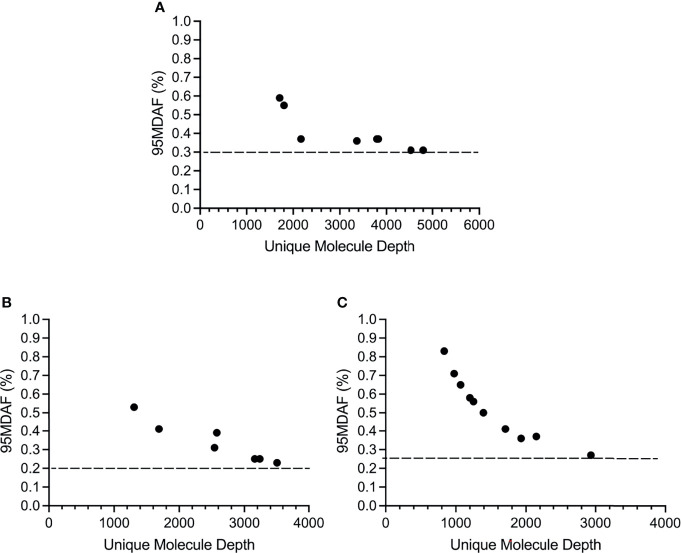
Sensitivity of the custom melanoma ctDNA panel based on BRAF, NRAS or *TERT* promoter mutations identified across the melanoma cohort. Each circle corresponds to the 95 minimal detectable allele fraction (95MDAF) values and unique molecule depth (unique fragments covering the specified region) for a single sample. **(A)** BRAF V600 mutations. Data derived from samples 1, 3, 4, 9, 10, 13, 16, 19. **(B)** NRAS G60/Q61 mutations. Data derived from samples 2, 4, 5, 6, 9, 11, 14. **(C)** TERT 228-250 promoter mutations. Data derived from samples 2, 4, 5, 6, 9, 13, 14, 16, 19, 20.

The cutoff for calling a mutation, based on the 95% confidence interval for the normal samples (95MDAF as defined in material and methods), of the custom melanoma ctDNA panel was inversely proportional to read depth but did approach a saturation point beyond which increasing read depth did not lead to a lower 95MDAF value (**Figure 3**). This 95MDAF value was ~0.3% for BRAF, 0.26% for *TERT* promoter mutations and 0.2% for NRAS mutations (**Figure 3**). The custom panel was also able to detect a MAP2K1 H119Y mutation with an 95MDAF of 0.18% (**Table S2**). In addition, several cancer-associated mutations were noted whereby the MAF was <95MDAF but still >50MDAF as defined in material and methods (**Table S2**). For these mutations further validation would be required. To demonstrate the accuracy of these low MAF cutoff values does ultimately require running a dilution series of samples for each mutation consisting of known MAFs.

### Validation of the Custom Melanoma ctDNA Panel

**Figure 4:**
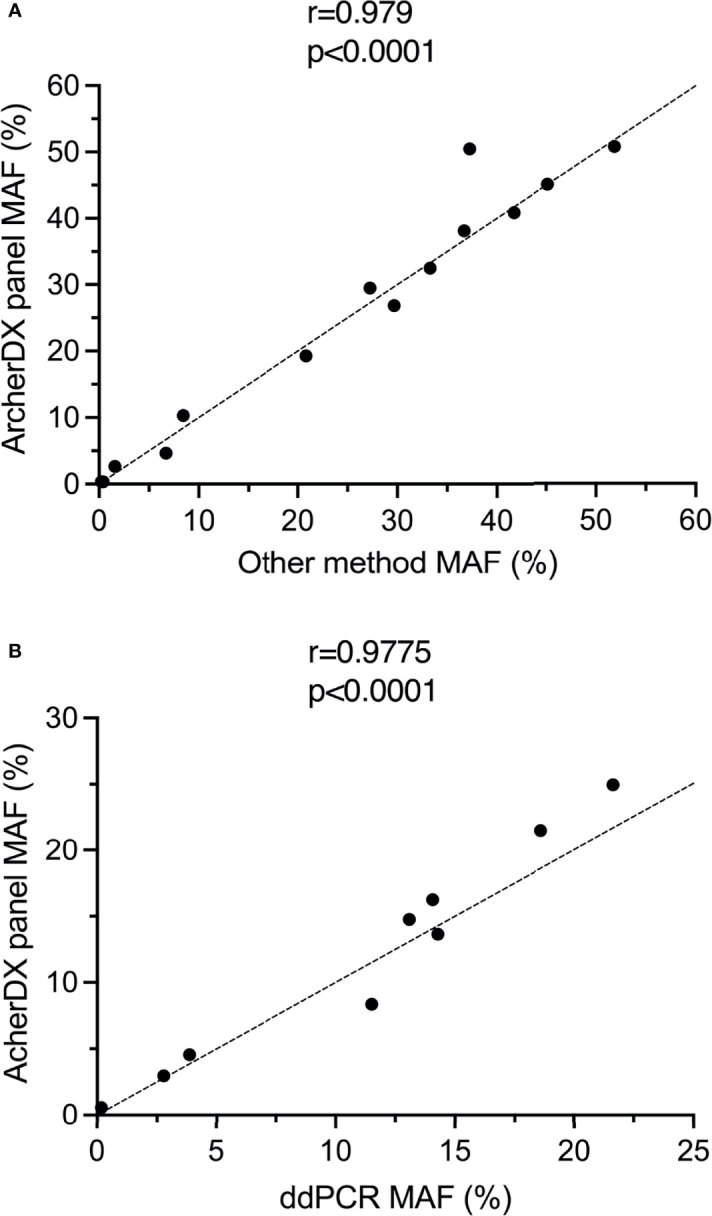
Validation of the ArcherDX custom melanoma ctDNA panel. **(A)** Correlation of driver mutant allele frequency (MAF) determined by the ArcherDX custom melanoma ctDNA panel versus other liquid biopsy analysis [ddPCR or Thermofisher custom melanoma ctDNA panel (25)]. **(B)** Correlation of *TERT* promoter MAF determined by the ArcherDX custom melanoma ctDNA panel versus ddPCR. For ddPCR data see **Table S3**. Pearson correlation coefficient analysis was performed.

All of BRAF V600E/K or NRAS Q61K/L/R driver mutations identified using the ArcherDX custom melanoma ctDNA panel and tissue biopsy were also identified using either ddPCR or a Thermofisher custom melanoma ctDNA panel (25) (**Table S2**). Furthermore, there was significant correlation in the MAF for each of these identified mutations when comparing the ArcherDX custom panel to these other liquid biopsy assays (**Figure 4A**). All of the *TERT* promoter C228T and C250T mutations identified with the custom panel were confirmed using ddPCR and showed significant correlation based on MAF (**Figure 4B**).

## Discussion

In this study, we have developed a custom melanoma NGS panel for detection of ctDNA. This panel was based on multiplex anchored PCR (51) from ArcherDX in contrast to previous studies which used either Thermofisher Ampliseq HD (25, 27), Qiagen QIAseq (24) or Illumina TruSeq Nano (28) workflows. An additional custom melanoma panel based on mass spectrometry detection has also been reported (29). None of these previously reported custom panels have successfully detected *TERT* promoter mutations, and our previous Thermofisher NGS panel yielded consistently low sequencing depth for the *TERT* promoter amplicons presumably due to the high GC content (>80%) (25). Given that *TERT* is one of the most frequently mutated genes in melanoma (42), the primary goal of our NGS panel design was to reliably detect the three common *TERT* promoter mutations in ctDNA.

The design of the current custom melanoma ctDNA panel was limited to key melanoma-associated gene mutations, but still had a theoretical coverage of ~81% of skin cutaneous melanoma [66% based on skin cutaneous melanoma TCGA database (45, 46) plus (15-60%) triple wild type melanoma with *TERT* promoter mutations (33, 38–40, 43, 44)]. Mutually exclusive somatic *TERT* promoter mutations, C250T, C228T and CC242TT, were found in 48% (10/21) of the melanoma cohort and increased the overall ctDNA detection rate from 67%, based on detection of driver (*NRAS* or *BRAF*) or cancer-associated mutations, to 71%. The limitation of the current study was that only four triple wild type samples were included. The custom melanoma panel detected only 50% of these four cases (based on detection of a *TERT* C250T mutation and a *MAP2K1* H119Y mutation respectively). Based on a *TERT* mutation frequency of 15-60% in triple wild type melanoma (33, 38–40, 43, 44) the *TERT* mutation detection rate of 25% of our wild type melanoma patients using this panel falls within the frequency range. A larger study incorporating many more triple wild-type melanoma samples is needed to accurately determine the detection sensitivity of this pilot ctDNA panel. We expect that analysis of a larger randomly selected or cross-sectional cohort would find more frequent *TERT* promoter mutations in the absence of *BRAF* or *NRAS* mutations, as previously reported (33, 38, 39). It is worth noting, however, that *TERT* promoter mutations may be subclonal and therefore under-represented in ctDNA relative to other driver mutations (24).

The overall detection rate of 71% in the current study was less than our previous custom ctDNA panel detection rate of 74% primarily due to a larger number of genes covered in our previous study (25). This was the case with lack of detection of BRAF G469E in one patient as this infrequent melanoma mutation (0.1% incidence) was not covered in the custom panel design. On the other hand, the custom panel did detect a rare NRAS G60V mutation in the same patient which was not detected by the tissue panel. This may possibly be due to the fact it is not a listed variant for tissue panels and therefore not called during analysis. A detectability of 20% in the stage III cohort was low although this was only based on five patient samples.

The ability to detect somatic *TERT* promoter mutations co-occurring with *BRAF* or *NRAS* mutations is also valuable given that a TERT-mutation positive genetic profile is associated with a worse prognosis for melanoma patients (30, 38, 79). Further reports suggest that *TERT* promoter mutations may be predictive of improved response to immunotherapy (80) and a poorer response to BRAF/MEK inhibition (35). Given our small cohort size and the fact that the majority of the patients were non-responders to immunotherapy, we were unable to conclude whether detection of *TERT* promoter mutations does in fact have any prognostic or predictive value.

We also detected the previously described germline single nucleotide polymorphisms (SNPs) in the TERT promoter T349C, G373A, G452C (70) in 81% of our melanoma cohort at a MAF ranging from 38 to 99%. The most frequent *TERT* SNP (T349C; rs2853669) was found in 76% of our melanoma cohort. This *TERT* SNP has been identified in a range of other cancers and may act to disrupt a pre-existing ETS binding site in the *TERT* promoter (81). Nevertheless, its prognostic role remains controversial with contrasting reports on its influence on *TERT* expression and differing conclusions on its prognostic value (43, 70–72, 82, 83, 85–88). In melanoma, *TERT* T349C has been reported to modify the effects of somatic *TERT* promoter mutations leading to increased survival in melanoma (40) and this may be mediated through a lengthening of telomeres (43). We found no obvious association of this variation either with somatic *TERT* mutation or patient outcomes in our melanoma cohort.

In addition to successful detection of *TERT* promoter mutations our current custom melanoma panel performed favorably, based on tissue concordance (76%), 95MDAF (0.2%) and significant correlation with ddPCR, when compared to previously published custom panels (24, 25, 27–29). Future studies will incorporate our findings from previous (25) and current panel designs to produce an optimized melanoma custom ctDNA panel with detection rates of at least 90% in stage IV cutaneous melanoma patients. An optimized melanoma ctDNA (50 gene targets), which will increase the theoretical coverage will be useful for monitoring residual disease in stage III patients after resection and therapy and for longitudinal monitoring of progression in stage IV melanoma patients. In both cases ctDNA mutations identified pre-treatment will be monitored longitudinally using targeted approaches such as ddPCR. To improve ctDNA detection sensitivity ctDNA mutation detection may also be complemented with ctDNA methylation analysis. Ultimately establishing if our optimized melanoma panel truly reaches the theoretical coverage will depend on analysis of a larger cohort consisting of 100-200 patients.

## Data Availability Statement

The original contributions presented in the study are publicly available. This data can be found here: https://www.ncbi.nlm.nih.gov/, PRJNA798432, https://www.ncbi.nlm.nih.gov/bioproject/PRJNA798432.

## Ethics Statement

The studies involving human participants were reviewed and approved by Sydney Local Health District Human Research Ethics Committee, Protocol No. X15–0454 and HREC/11/RPAH/444. Macquarie University Human Research Ethics Committee Protocol No. 52020195621941. The patients/participants provided their written informed consent to participate in this study.

## Author Contributions

Conceptualization, RD, JL, and HR. Methodology, RD and HR. Formal analysis, RD, JL, AS, and HR. Investigation, RD, JL, and AS. Resources, AM, MC, GL, RPMS, JS, and RAS. Writing—original draft preparation, RD and HR. Writing—review and editing, RD, JL, AS, AM, MC, GL, RPMS, JS, RAS, and HR. Visualization, RD, JL, and HR. Supervision, HR. Project administration, HR. Funding acquisition, HR. All authors contributed to the article and approved the submitted version.

## Funding

RD was supported in part by a donation to Melanoma Institute Australia from the CLEARbridge Foundation. This work was also supported in part by the National Health and Medical Research Council (APP1093017 and APP1128951). HR, RAS, and GL are supported by National Health and Medical Research Council Research Fellowships (APP1104503, APP1141295 and APP1119059). GL is also supported by the Medical Foundation of The University of Sydney and Melanoma Institute Australia. AMM is supported by a Cancer Institute NSW Fellowship, Nicholas and Helen Moore and Melanoma Institute Australia. JS and RPMS are supported by Melanoma Institute Australia. Support from colleagues at Melanoma Institute Australia, Royal Prince Alfred Hospital and Westmead Hospital is also gratefully acknowledged. Support from The Ainsworth Foundation is also gratefully acknowledged.

## Conflict of Interest

GL receives consultant service fees from Amgen, BMS, Array, Pierre-Fabre, Novartis, Merck Sharp & Dohme (MSD), Qbiotics, Roche and Sandoz. AM is an advisory board member for BMS, MSD, Novartis, Roche, Pierre Fabre and Qbiotics. MC is an advisory board member for MSD, BMS, Novartis, Pierre-Fabre, Roche and Amgen. RAS has received fees for professional services from Evaxion, Provectus Biopharmaceuticals Australia, Qbiotics Group Limited, Novartis Pharma AG, MSD Sharp & Dohme (Australia), NeraCare, AMGEN Inc., Bristol-Myers Squibb, Novartis Pharmaceuticals Australia Pty Limited, Myriad Genetics GmbH, GlaxoSmithKline Australia. RPMS has received honoraria for advisory board participation from MSD, Novartis and Qbiotics and speaking honoraria from BMS.

The remaining authors declare that the research was conducted in the absence of any commercial or financial relationships that could be construed as a potential conflict of interest.

## Publisher’s Note

All claims expressed in this article are solely those of the authors and do not necessarily represent those of their affiliated organizations, or those of the publisher, the editors and the reviewers. Any product that may be evaluated in this article, or claim that may be made by its manufacturer, is not guaranteed or endorsed by the publisher.
