# Supplementary figures and images for: Anchored Multiplex PCR Custom Melanoma Next Generation Sequencing Panel for Analysis of Circulating Tumor DNA
Source: Front Oncol. 2022 Apr 12;12:820510. doi: 10.3389/fonc.2022.820510 (PMC9039342; doi:10.3389/fonc.2022.820510)

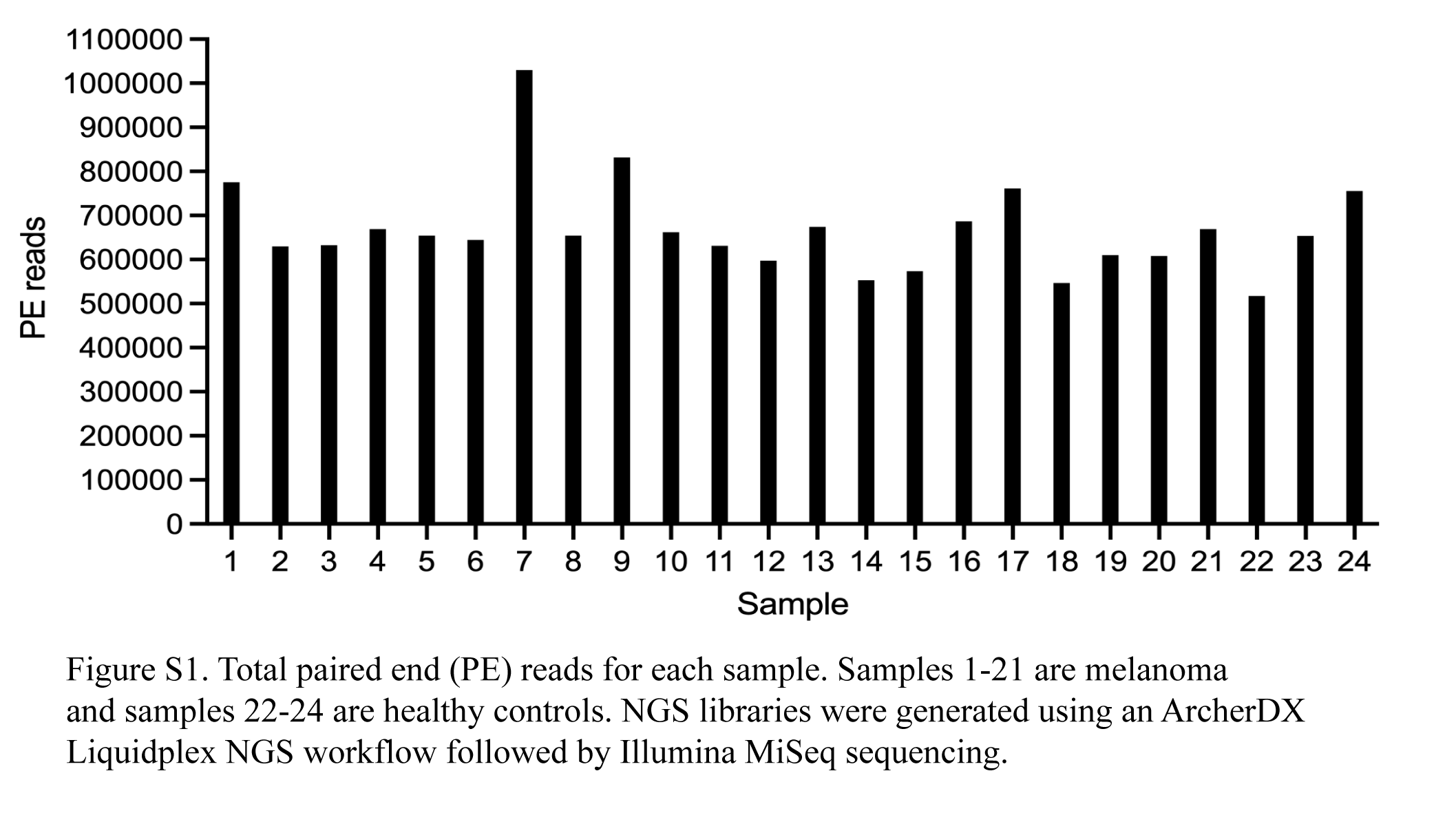

Supplement: Supplementary file 1 [file Image_1.tif]

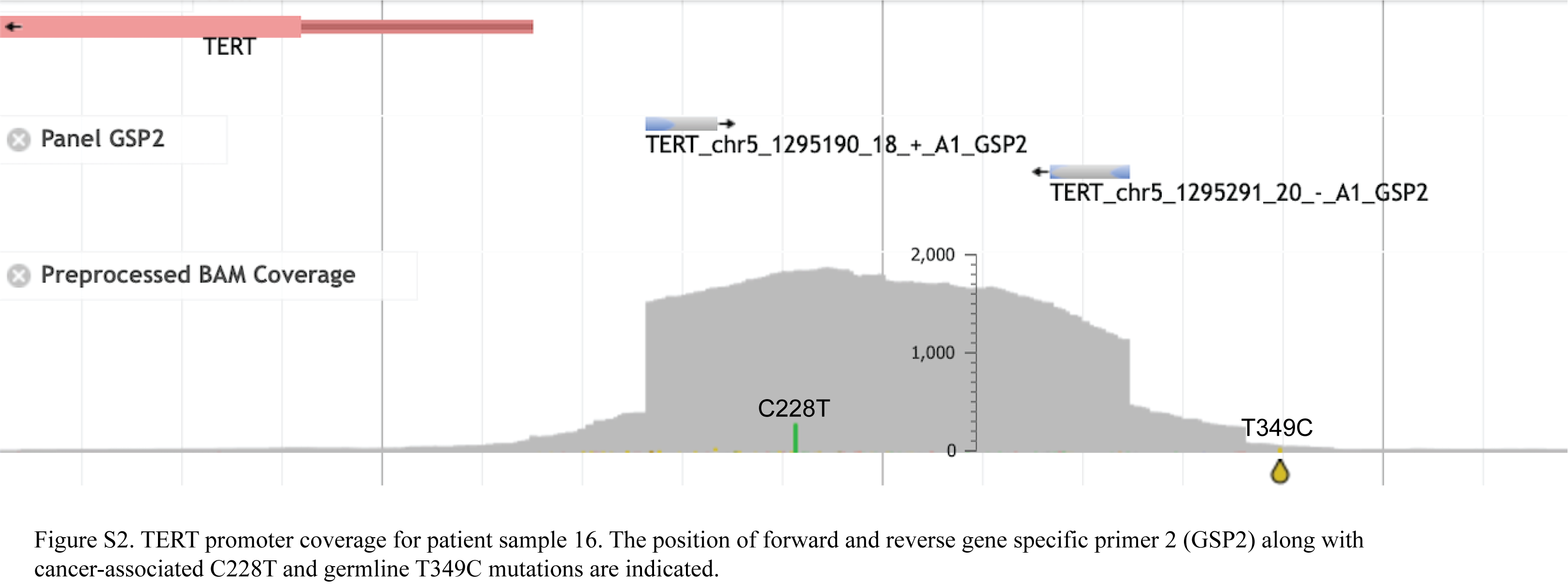

Supplement: Supplementary file 2 [file Image_2.tif]

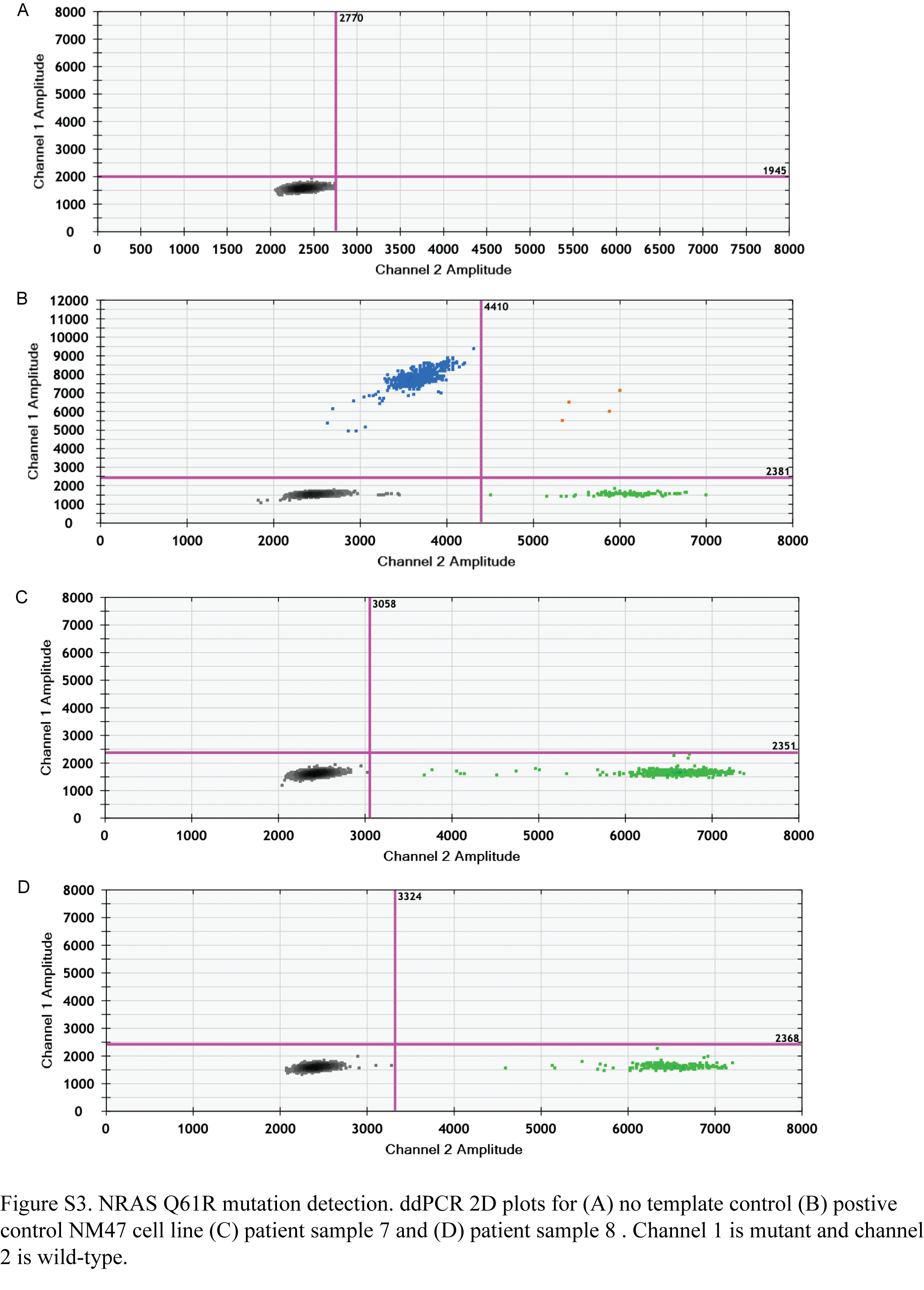

Supplement: Supplementary file 3 [file Image_3.tif]
